# Hospital Cases

**Published:** 1850-11

**Authors:** J. M. Steiner

**Affiliations:** Assistant Surgeon U. S. Army, Fort Graham, Texas


*Hospital Cases.* Reported by J. M. Steiner, M. D., Assistant Surgeon U. S. Army, Fort Graham, Texas.

Private White of "I" Company, 2d Dragoons, came into the Hospital, on the 11th of November, 1849, with the conjunctiva and cornea of the right eye severely inflamed. Constitution naturally strong, but depraved by vicious habits; complained of great pain in and around the orbit, and states that his eye became affected on the 6th. The inflammatory symptoms being high, he was bled copiously from the arm; cups were applied to the right temple; a solution of nit. argenti xx. grs. to the ounce of water, dropped in the eye, ter die; bowels freely moved, and a mercurial course adopted. Under this treatment the inflammation rapidly subsided. On the 25th the left eye became involved, but the inflammation disappeared in a few days, the nit. argenti alone being used. The eyes remained weak and irritable until the 20th of January, when the following complication occurred. From the appearance of the right eye, it seemed as if the crystalline lens had become detached on the inner side, from its attachment to the hyaloid membrane, which protruded into the anterior chamber, in the form of a small, semi-transparent pouch. He complained of a dull, aching pain, in and about the orbit, and the right temple. Several ounces of blood were abstracted by means of cups from the temple; blisters applied back of the ears, and the mercurial treatment resumed. On the 25th the pain had subsided; gums slightly touched; pouch gradually enlarging and assuming a hazy, or less transparent hue. Feb. 10th, anterior chamber nearly filled with pouch, which has assumed a milky color; vision almost entirely destroyed. From this time, until the 23rd of March, when the company to which he belongs, left this Post, no appreciable change took place. Among other medicines, the iodide of potassium was administered, but without benefit. If the hyaloid membrane has not become disorganized—which I believe to be the case—possibly an operation might effect a radical cure.

Private Edward Capon, of "H" Company, 8th Infantry, aged 31 years, of sanguine temperament, was received into the Hospital on the 3rd of March. He complained of violent pain, extending from the precordia, to the back, shoulders, and back of the neck, and running down the left arm as far as the elbow. There was considerable dyspnœa ; panting respiration ; fever, and a firm, full, sharp pulse, beating 115 per minute. The head, neck, and shoulders, drooped forward, and he was either unable or afraid to assume a straight position. On examining the lungs by auscultation and percussion, no morbid symptoms could be detected. Directing him to hold his breath, I applied my ear over the region of the heart, which I found to be the part affected. A rough grating, friction sound, could be distinctly heard, synchronous with the systole of the ventricles. No additional morbid sounds could be heard. The dulness in the region of the heart, did not appear to extend over a greater space than is usual in health. As this man had been affected several times, within the last twelve months, with acute articular rheumatism—the last attack occurring in the preceding month—I concluded his present complaint to be rheumatic carditis, or pericarditis. Fifteen ounces of blood were taken from the arm, and a dose of xx. grs. of calomel, with xv. grs. of Dover's powder administered. A purgative of senna and salts was given in the afternoon. At 9 P. M. cups were applied to the left side of the chest, and between the shoulders, and twelve ounces of blood procured. Ten grains of calomel, and one of opium were directed to be taken every six hours. The friction sound could still be heard on the 4th, but not so distinctly as on the day previous ; the febrile symptoms, had considerably abated ; the distressing dyspnœa lessened, and the pain moderated. Calomel and opium continued. On the 6th, there was no fever; pulse 96, irregular, and devoid of strength, pain slight ; the gums being touched by the calomel, it was discontinued and the impression kept up, by small doses of blue mass, given night and morning. On the 6th, a blister was applied over the region of the heart. On the 12th, he was able to walk about the wards. The cardiac symptoms, with the exception of the friction sound, recurred on the 24th, but in a milder form. He was relieved by cups, and a few doses of calomel, conjoined with the extract of aconite. At this date, March 31st, he is free from pain, and the sounds of the heart appear normal, though somewhat less distinct. I believe that the pericardium has become adherent to the heart. The patient is pale, weak, and emaciated, and I do not think will ever be completely restored to health.

Private Fink, of " A " Company, 2nd Dragoons, was received in the Hospital on the 15th of March, with a compound fracture of the bones of the right leg. The fracture was occasioned by a kick from a horse, as the man attempted to mount him. In falling the foot was bent backwards on the calf, and the upper fragment of the tibia, tore through the soft parts. On the 14th, the day previous to that on which the accident occurred, I had accompanied a scout into the Camanche Country, and did not return until the 18th; so that I did not see the patient till the 4th day after the receipt of the injury. The tibia was obliquely fractured, a little below its middle, and the lower end of the upper fragment denuded for three-fourths of an inch of the periosteum, protruded two inches and a half from a lacerated and contused wound in the soft parts, on the inner and anterior part of the leg; the fibula was fractured an inch and a half higher up, but did not appear externally. In consequence of the wound furnishing a ready outlet for the fluids, but little swelling had ensued; and the man being of a strong and robust constitution, I concluded to try and save the limb. I thought it advisable to remove that portion of the tibia divested of its investing membrane, and it was therefore excised, when the remaining portion was drawn within the wound, which was closed as far as practicable, by means of sutures and adhesive straps. The patient was placed on his right side, with the leg partially flexed resting on a pillow, and lint saturated with cold water, kept applied to the wound. The discharge from the wound consisted of serum, mixed with blood. Considering the extent of the injury, there was but little constitutional disturbance; the skin was hot; pulse full and strong, and the bowels torpid, but these symptoms abated after the operation of a brisk cathartic. On the 20th, the limb was placed in a fracture box, the flexed position changed to the extended, and the cold applications being agreeable to the patient, continued. The wound gradually healed by granulation, and on the 9th of April, the limb was placed in Dessault's apparatus for fractured thigh, and extension and counter extension made. The heel was placed in a padded ring, and daily bathed with a mixture of whiskey and water, with the view of preventing, if possible, excoriation, yet a bad sloughing sore ensued, which gave the patient considerable pain, and myself much annoyance; no other unpleasant symptoms occurred. The splints were removed on the 20th of May; the injured extremity is from a half to three fourths of an inch shorter than the sound one. The union of the tibia, is by bone, and without deformity. In consequence of the overlapping of the fractured ends of the fibula, firm union of that bone, had not, I think, taken place; and if it has not, the limb will scarce be of much practical use, till the fibula is cut down upon, and the overlapping portion excised. The patient left this post on the 23rd of June to join his Company at Fort Croghan.

August 16th. I have this day ascertained from the Captain of his company, that the trip from this post to Fort Croghan, excited inflammation between the fractured ends of the fibula, resulting in firm union.

